# Measures of excess $\dot{\mathrm{V}}$CO_2_ and recovery $\dot{\mathrm{V}}$CO_2_ as indices of performance fatigability during exercise: a pilot study

**DOI:** 10.1186/s40814-021-00840-0

**Published:** 2021-06-23

**Authors:** Liana C. Wooten, Brian T. Neville, Randall E. Keyser

**Affiliations:** 1 Department of Health, Human Function, and Rehabilitation Science, George Washington University, Washington, DC USA; 2 Department of Rehabilitation Science, College of Health and Human Services, George Mason University, Fairfax, VA USA; 3 Rehabilitation Medicine Department, National Institutes of Health Clinical Center, Bethesda, MD USA

**Keywords:** Performance fatigability, Excess $\dot{\mathrm{V}}$CO_2_, Recovery, Buffering, Cardiopulmonary exercise testing (CPET)

## Abstract

**Background:**

The severity of performance fatigability and the capacity to recover from activity are profoundly influenced by skeletal muscle energetics, specifically the ability to buffer fatigue-inducing ions produced from anaerobic metabolism. Mechanisms responsible for buffering these ions result in the production of excess carbon dioxide (CO_2_) that can be measured as expired CO_2_ ($\dot{\mathrm{V}}$CO_2_) during cardiopulmonary exercise testing (CPET). The primary objective of this study was to assess the feasibility of select assessment procedures for use in planning and carrying out interventional studies, which are larger interventional studies investigating the relationships between CO_2_ expiration, measured during and after both CPET and submaximal exercise testing, and performance fatigability.

**Methods:**

Cross-sectional, pilot study design. Seven healthy subjects (30.7±5.1 years; 5 females) completed a peak CPET and constant work-rate test (CWRT) on separate days, each followed by a 10-min recovery then 10-min walk test. Oxygen consumption ($\dot{\mathrm{V}}$O_2_) and $\dot{\mathrm{V}}$CO_2_ on- and off-kinetics (transition constant and oxidative response index), excess-$\dot{\mathrm{V}}$CO_2_, and performance fatigability severity scores (PFSS) were measured. Data were analyzed using regression analyses.

**Results:**

All subjects that met the inclusion/exclusion criteria and consented to participate in the study completed all exercise testing sessions with no adverse events. All testing procedures were carried out successfully and outcome measures were obtained, as intended, without adverse events. Excess-$\dot{\mathrm{V}}$CO_2_ accounted for 61% of the variability in performance fatigability as measured by $\dot{\mathrm{V}}$O_2_ on-kinetic ORI (ml/s) (*R*^2^=0.614; *y* = 8.474*x* − 4.379, 95% CI [0.748, 16.200]) and 62% of the variability as measured by PFSS (*R*^2^=0.619; *y* =  − 0.096*x* + 1.267, 95% CI [−0.183, −0.009]). During CPET, $\dot{\mathrm{V}}$CO_2_ -off ORI accounted for 70% (*R*^2^=0.695; *y* = 1.390*x* − 11.984, 95% CI [0.331, 2.449]) and $\dot{\mathrm{V}}$CO_2_ -off Kt for 73% of the variability in performance fatigability measured by $\dot{\mathrm{V}}$O_2_ on-kinetic ORI (ml/s) (*R*^2^=0.730; *y* = 1.818*x* − 13.639, 95% CI [0.548, 3.087]).

**Conclusion:**

The findings of this study suggest that utilizing $\dot{\mathrm{V}}$CO_2_ measures may be a viable and useful addition or alternative to $\dot{\mathrm{V}}$O_2_ measures, warranting further study. While the current protocol appeared to be satisfactory, for obtaining select cardiopulmonary and performance fatigability measures as intended, modifications to the current protocol to consider in subsequent, larger studies may include use of an alternate mode or measure to enable control of work rate constancy during performance fatigability testing following initial CPET.

## Key messages regarding feasibility

- This pilot study fulfilled its objective of assessing the feasibility of the current study protocol, using select measurements, for future larger interventional studies investigating the relationships between CO_2_ expiration, measured during and after both CPET and submaximal exercise testing, and performance fatigability.
- While the current study demonstrated that utilizing the 10-MWT is achievable, alternatives to the PFSS using the 10-MWT as a primary outcome measure warrant consideration in future study.
- The findings of the current study suggest that utilizing the current protocol may produce measures of $\dot{\mathrm{V}}$CO_2_ as a novel laboratory measurement of performance fatigability.

## Background

The ability to sustain all physical activity is dependent on the energy substrate provided by oxidative phosphorylation [6, 18, 22]. If oxidative capacity is insufficient for meeting this demand entirely or if buffering of the ionic by-products of anaerobic metabolism is insufficient for maintaining an optimal intracellular pH, a competitive environment emerges in which an increase in glycolytic by-product accumulation tends to inhibit cross-bridge formation and metabolic pump activity [6, 18]. Gross muscle function impairment, reduced cardiorespiratory capacity, decreased exercise tolerance, and increased performance fatigability follow [13, 20, 32, 33]. Mechanisms such as lactate formation and the carbonic anhydrase-bicarbonate system buffer these fatigue-inducing hydrogen ions (H^+^) resulting in what is often called “non-metabolic or excess carbon dioxide (CO_2_)” production [4, 6, 22]. This accumulation can be observed in concomitance with a departure of the rise of expired CO_2_ ($\dot{\mathrm{V}}$CO_2_) plotted on oxygen consumption ($\dot{\mathrm{V}}$O_2_) or time from linearity during a cardiopulmonary exercise test (CPET) [4]. The $\dot{\mathrm{V}}$CO_2_ deflection point is designated by the terms anaerobic threshold (AT), gas exchange threshold, or ventilatory threshold although other terms may also be appropriate, and all are often used interchangeably.

Previous studies have associated $\dot{\mathrm{V}}$O_2_ recovery kinetics with survival and disease severity, in addition to serving as an index of functional capacity in subsets of apparently healthy individuals and in clinical population subsets [10, 14, 27]. Associations of performance fatigability with $\dot{\mathrm{V}}$O_2_ off-kinetics following peak and submaximal exercise has also been demonstrated [39]. While the recovery phase following activity appears to be an aerobic process, the $\dot{\mathrm{V}}$CO_2_ response during recovery has been suggested to be guided by the degree of anaerobic by-product accumulation and the rate at which the by-product is dissipated during the exercise bout [26]. Thus, the return to baseline metabolic homeostasis may also be inhibited collinearly with the magnitude of decrease in pH and the clearance of H^+^ accumulated during the activity [6, 22].

Previous studies on performance fatigability have identified significant relationships between measures of aerobic capacity such as peak oxygen consumption [33] (peak-$\dot{\mathrm{V}}$O_2_) and performance measures such as timed-walk test results and CPET duration [3, 20, 21, 31, 34, 36]. Additionally, oxygen uptake kinetics (VO_2_ on-kinetics) has been utilized as a marker of exercise tolerance or performance fatigability [8, 15, 16]. A possible relationship between excess CO_2_ expiration and running performance [17] has also been suggested and concurrent increases in the time (AT-time) required to achieve the anaerobic threshold (expressed as relative $\dot{\mathrm{V}}$O_2_; AT-$\dot{\mathrm{V}}$O_2_), distance attained on a timed walk test, and improvement peak and submaximal $\dot{\mathrm{V}}$O_2_/work rate ratio [20] following aerobic exercise training, even though no significant improvement in peak oxygen consumption was observed, have been reported. Moreover, AT-$\dot{\mathrm{V}}$O_2_ has been indirectly associated with Fatigue Severity Scale scores and the ability to meet energy requirements of instrumental activities of daily living [19]. However, $\dot{\mathrm{V}}$CO_2_ relationships have been less frequently considered and are less well understood.

As recently proposed by Severin and Gurovich [35], further understanding of basic and applied physiological concepts is an integral component and a basis of transformation for clinical practice. Previous research has underscored the importance of understanding relationships among functional capacity and cardiopulmonary function since these often used clinical trial outcome measures that have been shown to reflect longevity and physical activity tolerance [23, 29] and health-related quality of life [7]. Additionally, measures of $\dot{\mathrm{V}}$CO_2_ obtained during exercise testing protocols, in relation to functional capacity and cardiorespiratory function and exercise tolerance, are underutilized and understudied. In advance of a larger scale study, as measures of $\dot{\mathrm{V}}$CO_2_ during and post exercise are seldom assessed as primary outcomes, this study was designed to assess the integrity of utilizing the current study protocol to obtain these measures as well as measures of performance fatigability. Thus, the primary objective of this study was to assess the feasibility of the current study protocol for future, larger interventional studies investigating the relationships between CO_2_ expiration, measured during and after both CPET and submaximal exercise testing, and performance fatigability.

## Methods

### Inclusion/exclusion criteria

Healthy individuals between the ages of 18 and 45 were recruited for this study. Inclusion criteria included having a body mass index (BMI) of <35 and the ability to speak English fluently. Exclusion criteria included history or present symptoms of ischemic heart disease, left-sided heart failure, cor pulmonale or pulmonary hypertension, dilated hypertrophic cardiomyopathy or non-idiopathic cardiomyopathy; significant pulmonary dysfunction, e.g., COPD or ILD; hypertension defined as >160/90 mmHg; significant hepatic or renal dysfunction; chronic anticoagulation with warfarin or history of bleeding disorder; history or presence of any form of cancer other than skin cancer; stroke; active substance abuse or severe psychiatric disease; HIV infection; medications that limit exercise capacity or the ability to adapt to aerobic exercise training (e.g., beta blockers, antiretrovirals); diabetes mellitus; mitochondrial disease; presence of autoimmune, musculoskeletal, or neuromuscular disease; smoking; known pregnancy; anemia (hematocrit < 35%); or fibromyalgia. Exclusion criteria also include the history of any condition or current use of any medication that would make participation unsafe or alter performance or outcome of the protocol. This protocol was reviewed and approved by the George Mason University institutional review board and informed consent was obtained from all of the subjects prior to participation.

### Procedures

Subjects were asked to visit the Rehabilitation Science Exercise Physiology lab at George Mason University on two separate occasions, with a minimum of 48 h and maximum of 7 days in between each visit. During visit one, subjects underwent peak treadmill CPET followed by a 10-min recovery period and a 10-min walk test (10-MWT) immediately following the recovery period. The CPET was performed to obtain measures of cardiorespiratory fitness, total excess $\dot{\mathrm{V}}$CO_2_, and $\dot{\mathrm{V}}$O_2_ and $\dot{\mathrm{V}}$CO_2_ off-kinetics.

During visit two, subjects completed a submaximal constant work rate test (CWRT) again followed by a 10-min recovery period and a 10-MWT immediately following the recovery period. The CWRT was a constant square wave test in which subjects walked for 6 min at a work-rate corresponding to 80% of their anaerobic threshold (AT), the point during exercise in which there is increased reliance on anaerobic metabolism to support aerobic metabolism, determined from each subject’s peak CPET. Following this 6-min bout, subjects had an 8-min active recovery period. Subjects completed 3 cycles of this combination with a 10-min passive recovery following the third 6-min active bout during which recovery $\dot{\mathrm{V}}$O_2_ and $\dot{\mathrm{V}}$CO_2_ off-kinetics were measured. $\dot{\mathrm{V}}$O_2_ on-kinetics was determined as an ensemble average at 80% AT over the three 6-min exercise periods.

Subsequent to both the CPET and CWRT, subjects performed a 10-MWT following in order to obtain PFSS. The method developed by Schnelle and coworkers was used for these measures [34]. Specifically, subjects were asked to walk as far as they could within the 10-min period and running was not permitted. Total time taken between the end of the 10-min passive recovery period following both CPET and CWRT (during which data were obtained for the recovery kinetics) and the start of the 10-MWT was minimized for all subjects for consistency.

During both CPET and CWRT, cardiopulmonary function was assessed by pulmonary gas exchange analysis (MedGraphics CardiO2 CPET system, Medical Graphics Corporation, St Paul, MN). The gas analysis system was calibrated according to manufacturer’s specifications prior to each CPET and CWRT. During the CPET, treadmill workload was advanced in 3-min intervals according to the Bruce protocol. Heart rate (HR) was measured continuously by electrocardiogram (EKG). The endpoint for the CPET was decided a priori as volitional exhaustion defined as the subject’s indication that she/he must stop due to severe fatigue, despite significant encouragement by the investigational team to proceed.

### Determination of variables

#### Traditional $\dot{\mathrm{V}}$O_2_ indices

Peak $\dot{\mathrm{V}}$O_2_ was determined by an 8-breath average at the end of the test or at the end of the last completed stage, whichever was higher. The AT, a marker denoting the onset of exercise-induced fatigue based on expired carbon dioxide and other gas exchange variables during the CPET, was determined using the V-slope method [4] applied to the breath-by-breath measurements and reported as AT-$\dot{\mathrm{V}}$O_2_.

#### Kinetics

Pulmonary gas exchange was analyzed breath-by-breath continuously throughout the test. $\dot{\mathrm{V}}$O_2_ on-transient response, obtained during the CWRT, was modeled using nonlinear, least squares regression fitting techniques (Origin, OriginLab Corp., Northhampton, MA, USA) with a mono-exponential function of the form:

$$ \dot{\mathrm{V}}{\mathrm{O}}_2\left(\mathrm{t}\right)=\left(\Delta \dot{\mathrm{V}}{\mathrm{O}}_{2\mathrm{baseline}}\right)+\mathrm{Amplitude}\left(1-{\mathrm{e}}^{-\left(\mathrm{t}-\mathrm{TD}\right)/\uptau}\right) $$

where $\dot{\mathrm{V}}$O_2_ (t) represents $\dot{\mathrm{V}}$O_2_ as a function of time (t) throughout the exercise transient; $\dot{\mathrm{V}}$O_2_ baseline is the baseline $\dot{\mathrm{V}}$O_2_ data collected immediately prior to start of the exercise test; Amplitude is the amplitude increase in $\dot{\mathrm{V}}$O_2_ above the baseline value; tau (*τ*) is the time constant, or the time taken to reach 63% of the steady-state response; and TD is the time delay [9]. $\dot{\mathrm{V}}$CO_2_ off-kinetic response following the CWRT utilized the same model substituting $\dot{\mathrm{V}}$CO2 in place of $\dot{\mathrm{V}}$O2, steady state in place of baseline, and Amplitude was the amplitude during recovery. 

$\dot{\mathrm{V}}$O_2_ off-kinetics following CPET was determined using the formula similar to that used by Ozyener et al. [28].

$$ \dot{\Delta \mathrm{V}}{\mathrm{O}}_2\ \left(\mathrm{t}\right)=\dot{\mathrm{V}}{\mathrm{O}}_{2\mathrm{baseline}}+{\mathrm{Ae}}^{-\left(\mathrm{t}-\mathrm{TD}\right)/\uptau}\kern0.5em $$

Where $\dot{\mathrm{V}}$O_2baseline_ in this case is the $\dot{\mathrm{V}}$O_2_ at baseline recovery. As the initial “cardiodynamic” phase of the kinetic response is not well understood during recovery, the first 20 s was not included in the fit [28]. All fits were made to the end of the 10-min recovery period (i.e., 600 s from start of recovery) and optimized by minimization of the residuals around the *Y* axis (*Y*= 0) and sum of squares. $\dot{\mathrm{V}}$CO_2_ off-kinetics was determined using the same model substituting $\dot{\mathrm{V}}$CO_2_ values and iterations in place of $\dot{\mathrm{V}}$O_2_. From these models, a mean response time (MRT) was estimated as the sum of tau and the time delay. The transition constant (Kt) was calculated as the Δ$\dot{\mathrm{V}}$O_2_/MRT and the oxidative response index (ORI) was calculated as the Δ$\dot{\mathrm{V}}$O_2_/tau, both calculations utilized to normalize the response time to the amplitude [19].

#### Performance fatigability severity

PFSS were calculated using the method of Schnelle [34]. During the 10-MWT, walking velocities at 2.5 min and at the end of the 10-min test were calculated. The ratio of change in walking speed from the first 2.5-min interval to walking speed over the entire distance, normalized to the total distance walked over the test was used to calculate the performance fatigability severity score. The formula used was as follows:

$$ \frac{\left(\mathrm{speed}\ \left(\mathrm{m}/\mathrm{s}\right)\ \mathrm{at}\ 10\ \mathrm{minutes}/\mathrm{s}\mathrm{peed}\ \left(\mathrm{m}/\mathrm{s}\right)\ \mathrm{at}\ 2.5\ \mathrm{minutes}\right)}{\mathrm{total}\ \mathrm{distance}\ \mathrm{walked}\ \left(\mathrm{m}\right)} $$

Scores were multiplied by 1000 for reporting purposes.

#### Total excess $\dot{\mathrm{V}}$CO_2_

**Fig. 1:**
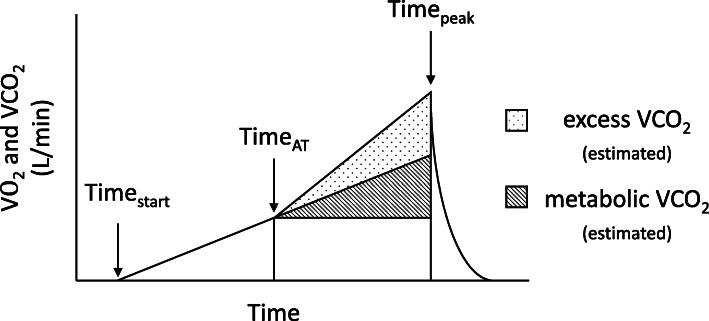
Schematization of $\dot{\mathrm{V}}$CO_2_ and its energy repletion (metabolic $\dot{\mathrm{V}}$CO_2_) and buffering (excess $\dot{\mathrm{V}}$CO_2_) components

Excess $\dot{\mathrm{V}}$CO_2_ was measured by calculating the difference between estimated total area of $\dot{\mathrm{V}}$CO_2_ and estimated area of metabolic $\dot{\mathrm{V}}$CO_2_ above the anaerobic threshold (Fig. 1).

- Total $\dot{\mathrm{V}}$CO_2_ was calculated using the following formula:
  $$ \left[\left(\mathrm{peak}\ \mathrm{time}-\mathrm{AT}\ \mathrm{time}\right)\times \left(\mathrm{peak}\ \dot{\mathrm{V}}\mathrm{CO}2-\mathrm{AT}\ \dot{\mathrm{V}}\mathrm{CO}2\right)\right]/2 $$
- Metabolic $\dot{\mathrm{V}}$CO_2_ was estimated using the same formula but first calculating the estimated peak metabolic $\dot{\mathrm{V}}$CO_2_ (using the slope of $\dot{\mathrm{V}}$CO_2_ line from time zero to the anaerobic threshold and extending to peak test duration time in seconds) and substituting this value in for peak $\dot{\mathrm{V}}$CO_2_.
- Excess-$\dot{\mathrm{V}}$CO_2_ was estimated by calculating the difference between total $\dot{\mathrm{V}}$CO_2_ and metabolic $\dot{\mathrm{V}}$CO_2_ and converting to liters.

#### Variables and statistics

Cardiopulmonary and performance fatigability measures consisted of excess-$\dot{\mathrm{V}}$CO_2_, performance fatigability as measured by PFSS (obtained during the 10-min walk test) and $\dot{\mathrm{V}}$O_2_ on-kinetics, and recovery indices (off-kinetics ORI, and Kt). Additional variables of interest were those characterizing the cardiorespiratory response during the peak CPET including $\dot{\mathrm{V}}$O_2_-peak, AT-$\dot{\mathrm{V}}$O_2_, peak time, and AT-time. Data were analyzed using regression analyses in Stata version 14.2 (StataCorp, College Station, TX). Regression equations and 95% confidence intervals for the respective independent variable coefficient are reported.

#### Feasibility outcomes

Feasibility of the current study was assessed based on criteria separated into three categories, (1) recruitment and retention; (2) procedures and measures; and (3) preliminary evaluation of participant data. Each category was evaluated following completion of the study to determine the success of the current feasibility study for informing a future large-scale study.

## Results

Seven apparently healthy subjects, 5 females and 2 males, participated in the cross-sectional, pilot study (Table 1) between November and December of 2017. All subjects that met the inclusion/exclusion criteria and consented to participate in the study completed all exercise testing sessions with no adverse events. Furthermore, all testing procedures were carried out and outcome measures obtained successfully, as intended.

**Table 1:** Subject demographics

	Age (years)	Sex	Height (cm)	Weight (kg)	BMI (kg/m^2^)
Subject					
1	28	M	182	114	34
2	30	F	159	60	24
3	28	F	158	63	25
4	40	F	163	61	23
5	31	M	177	101	32
6	34	F	170	68	24
7	24	F	170	54	19
Mean ± SD	31 ± 5.1		168 ± 9.2	74 ± 23.1	25.9 ± 5.5
Median	30		170	63	24
Range	24‑40		158‑182	54‑114	19‑34

*BMI* Body mass index

Data presented by individual subject. Mean data presented as mean ± 1 standard deviation unit

During the peak CPET, all subjects attained a respiratory exchange ratio (RER) of at least 1.10 and a peak HR of at least 90% of their age-predicted values (Table 2), indicating a level of exertion that elicited a physiologically maximal metabolic demand at volitional exhaustion [2]. Resting, AT, and peak exercise data including HR, RER, time, $\dot{\mathrm{V}}$O_2_, and $\dot{\mathrm{V}}$CO_2_ are reported in Table 2 along with PFSS.

**Table 2:** Resting, AT, and peak exercise data including HR, RER, time, $\dot{\mathrm{V}}$O_2_, and $\dot{\mathrm{V}}$CO_2_ along with PFSS

	Resting	AT	Peak
	Mean ± SD Median Range		
**HR (bpm)**	86 ± 7 (80, 92)	133 ± 12 (122, 144)	188 ± 7 (182, 194)
**RER**	0.90 ± 0.05 (0.86, 0.94)	0.82 ± 0.02 (0.80, 0.85)	1.20 ± 0.80 (1.13, 1.28)
$\dot{\mathbf{V}}$**O**_**2**_ (ml/(kg*min))	4.6 ± 0.7 (3.97, 5.32)	20.9 ± 2.8 (18.3, 23.4)	35.6 ± 4.7 (31.2, 40.0)
$\dot{\mathbf{V}}$**O**_**2**_ **(ml/min)**	345 ± 117 (237, 453)	1594 ± 579 (1059, 2130)	2915 ± 854 (2126, 3705)
$\dot{\mathbf{V}}$**CO**_**2**_ **(ml/min)**	310 ± 106 (212, 409)	1315 ± 483 (868, 1761)	3522 ± 1025 (2575, 4470)
**Time (s)**		223 ± 50 (177, 270)	580 ± 83 (504, 657)
**PFSS**^a^	0.98 ± 0.14 (0.86, 1.11)		

*AT* Anaerobic threshold, *HR* Heart rate, *RER* Respiratory exchange ratio, $\dot{V}$*O*_*2*_ Oxygen consumption, $\dot{V}$*CO*_*2*_ Expired carbon dioxide, *PFSS* Performance fatigability severity score

Data presented as mean ± 1 standard deviation unit and (95% confidence interval)

^a^Performed following exercise testing

**Fig. 2:**
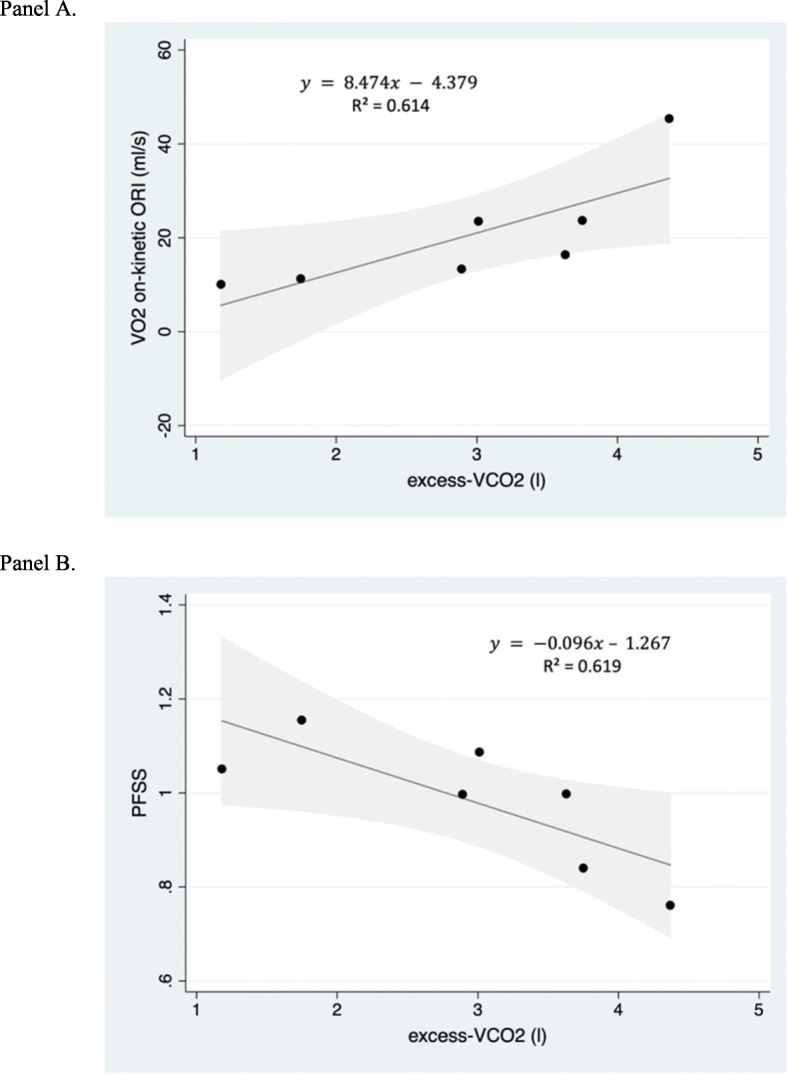
Excess-$\dot{\mathrm{V}}$CO_2_ and performance fatigability. **a** Total expired excess carbon dioxide (excess-$\dot{\mathrm{V}}$CO_2_) as a predictor of performance fatigability. *X*-axis represents $\mathrm{excess}\ \dot{\mathrm{V}}$CO_2_ in liters. *Y*-axis represents performance fatigability as measured by $\dot{\mathrm{V}}$O_2_ on-kinetic ORI in ml/s. Regression equation and *R*^2^ values reported. Gray-shaded area indicates 95% confidence intervals. **b** Total expired excess carbon dioxide (excess-$\dot{\mathrm{V}}$CO_2_) as a predictor of performance fatigability. *X*-axis represents nm-$\dot{\mathrm{V}}$CO_2_ in liters. *Y*-axis represents performance fatigability as measured by PFSS (performance fatigability severity score). Regression equation and *R*^2^ values reported. Gray-shaded area indicates 95% confidence intervals

As shown in Fig. 2, excess-$\dot{\mathrm{V}}$CO_2_ accounted for 61% of the variability in performance fatigability as measured by $\dot{\mathrm{V}}$O_2_ on-kinetic ORI (ml/s) (*R*^2^=0.614; *y* = 8.474*x* − 4.379, 95% CI [0.748, 16.200]) and 62% of the variability as measured by PFSS (*R*^2^=0.619; *y* =  − 0.096*x* + 1.267, 95% CI [−0.183, −0.009]). During CPET, $\dot{\mathrm{V}}$CO_2_-off ORI accounted for 70% (*R*^2^=0.695; *y* = 1.390*x* − 11.984, 95% CI [0.331, 2.449]) and $\dot{\mathrm{V}}$CO_2_-off Kt for 73% of the variability in performance fatigability measured by $\dot{\mathrm{V}}$O_2_ on-kinetic ORI (ml/s) (*R*^2^=0.730; *y* = 1.818*x* − 13.639, 95% CI [0.548, 3.087]). During sub-maximal CWRT, $\dot{\mathrm{V}}$CO_2_-off ORI accounted for 93% (*R*^2^=0.928; *y* =  − 0.956*x* + 4.493, 95% CI [−1.262, − 0.649]) and $\dot{\mathrm{V}}$CO_2_-off Kt for 96% of the variability in performance fatigability (*R*^2^=0.955; *y* =  − 1.101*x* + 3.883, 95% CI [−1.376, − 0.825]) in this sample. $\dot{\mathrm{V}}$CO_2_-off kinetics ORI following peak CPET also accounted for 57% of the variance as measured by PFSS (*R*^2^=0.566; *y* =  − 0.014*x* + 1.316, 95% CI [−0.028, 0]). In contrast, measures of $\dot{\mathrm{V}}$O_2_ obtained during exercise testing accounted for only 39% (peak relative $\dot{\mathrm{V}}$O_2_: *R*^2^=0.388; *y* = 1.605*x* − 36.607, 95% CI [−0.715, 3.925]; (AT-$\dot{\mathrm{V}}$O_2_: *R*^2^=0.297; *y* = 2.432*x* − 30.272, 95% CI [−1.867, 6.731]) of performance fatigability in this sample as measured by $\dot{\mathrm{V}}$O_2_ kinetics. However, AT-$\dot{\mathrm{V}}$O_2_ accounted for 82% (*R*^2^=0.818; *y* =  − 0.046*x* + 1.937, 95% CI [−0.070, −0.021]) and peak $\dot{\mathrm{V}}$O_2_ for 73% (*R*^2^=0.731; *y* =  − 0.025*x* + 1.872, 95% CI [−0.042, −0.008]) of PFSS in this sample.

## Discussion

The findings of the current study support the use of the current protocol, with modifications, for future larger studies investigating relationships between measures of $\dot{\mathrm{V}}$CO_2_ and performance fatigability. Methods were found to be satisfactory, including criteria for recruitment, retention, general structure of testing days, and peak CPET protocol. Based on the current study, reconsideration of the performance fatigability field test for more favorable alternative measures may be warranted. In the current study, the 10-MWT was used to obtain performance fatigability severity scores, one of the primary outcomes for performance fatigability. While using a field test such as the 10-MWT to obtain PFSS scores was feasible and may be more clinically applicable, results can be impacted by subject motivation and the inability to hold work rate constant, which makes comparison between individuals more difficult. Additionally, the experience with these relatively young, healthy adults suggests an alternative measure may be more sensitive, since Schnelle’s measure was validated in older adults and may be more appropriate for that population (age 65+ years). The use of $\dot{\mathrm{V}}$O_2_ kinetics was also utilized in this current pilot study as a measure of performance fatigability. While measures of $\dot{\mathrm{V}}$O_2_ on-kinetics provide an objective measure that is not influenced by subject motivation, it also lacks information regarding the ability of an individual to sustain a given work rate, especially at higher intensities, or work rates above the anaerobic threshold.

Additionally, a larger study would be improved by narrowing down primary objectives for performance fatigability to one primary outcome measure, such as time to fatigue during a peak CPET. Furthermore, we offer that there may be additional advantages to utilizing a stationary cycling-based peak CPET and subsequent fatigability testing. Advantages to such an alternative configuration include the ability to continue monitoring cardiopulmonary measures and more accurate quantification of work, as well as the ability to structure a constant work rate during such testing, which was not possible utilizing the 10-MWT field test in the current study. Additional measures of cardiopulmonary function may also be more easily collected during a cycle-based set of testing and would provide additional insight. While we agree that there are advantages to investigating ambulatory measures, the cost and accuracy of portable metabolic equipment are barriers to such field testing protocols currently. This pilot provides initial findings that measures of $\dot{\mathrm{V}}$CO_2_ may provide acceptable, novel laboratory measurements of performance fatigability manifestation, such as recovery $\dot{\mathrm{V}}$CO_2_ kinetics following submaximal exercise, explained over 90% of the variance in most of the performance fatigability outcomes. Thus, in summary, feasibility objectives were met and support the use of the current protocol, with modifications, for future larger studies investigating relationships between measures of $\dot{\mathrm{V}}$CO_2_ and performance fatigability.

It should be noted that the current study intended to demonstrate use of the current protocol to determine its feasibility and did not aim to delineate primary mechanisms for the sequence of biochemical events leading to increased $\dot{\mathrm{V}}$CO_2_ during more strenuous exercise and during the post-exercise recovery period. However, intensity dependent accumulation of by-products interferes with the ability to sustain physical activity while increasing $\dot{\mathrm{V}}$CO_2_ above that occurring directly with increases in Krebs Cycle activity and oxidative metabolic function. Hirakoba et al. [17] found that an absolute change in excess CO_2_, relative to mass and plasma lactate, was significantly related to the absolute change in distance running performance following endurance training. In the current study, the total expired non-metabolic CO_2_ (excess-$\dot{\mathrm{V}}$CO_2_) was observed to be strongly associated with measures of performance fatigability and the rate at which $\dot{\mathrm{V}}$CO_2_ returned to pre-exercise homeostasis following the submaximal exercise perturbation was the most strongly correlated with measures of performance fatigability.

As a relatively new construct, the underlying mechanisms and functional limitations associated with fatigability are not completely understood. However, fatigue affects all individuals regardless of age, gender, or health status creating debilitating effects on physical function [1, 5, 12, 30, 37] and is one of the most common complaints of individuals seen in primary care settings [5]. Furthermore, in older adults, fatigue has been shown to create significant health implications as it is associated with poorer mobility, functional limitations, disability, and mortality [21, 25, 36]. Although it is thought to be more commonly associated with diagnosed medical conditions, only one-third of all fatigue complaints can be attributed to disease [37]. Improving our understanding of the mechanisms underlying fatigability and our ability to more specifically measure its impact on physical activity may help shape the way health practitioners approach fatigue management and improve our ability to provide effective clinical interventions.

### Limitations

The small, non-randomized, convenience sample utilized in this pilot could limit generalizability and interpretations that may be drawn from the statistical analyses of these data. The 10-MWT utilized in order to obtain PFSS has previously been validated in older adults, but to our knowledge, this field test has not been validated in younger populations. Furthermore, the inability to control constancy of work rate throughout this type of test is a limitation that may be remedied with alternative testing protocols. Non-metabolic or excess $\dot{\mathrm{V}}$CO_2_ cannot currently be differentiated through direct measurement and thus must be estimated from an algorithm. In the current study, the excess CO_2_ produced from the buffering of H^+^ ions was differentiated algebraically from the CO_2_ produced as a result of normal metabolic processes. The calculation also assumes that progression from AT-$\dot{\mathrm{V}}$CO_2_ to peak $\dot{\mathrm{V}}$CO_2_ remains linear. Underlying the calculation is the generally accepted assumption that $\dot{\mathrm{V}}$CO_2_ would continue rising linearly with $\dot{\mathrm{V}}$O_2_ throughout exercise if the volume of excess $\dot{\mathrm{V}}$CO_2_ above AT was not included in the response. AT was determined by the V-slope method, which is based on simultaneous $\dot{\mathrm{V}}$O_2_ and $\dot{\mathrm{V}}$CO_2_ measurements that are not independent of the subject’s ventilatory response and sensitive to individual breathing irregularities. Moreover, plasma lactate, bicarbonate, and pH were not measured, so collinearity could not be determined with respect to these measures of acidemia and $\dot{\mathrm{V}}$CO_2_. Conversely, Zoladz and coworkers ([40]) observed that the critical power for both $\dot{\mathrm{V}}$O_2_ and $\dot{\mathrm{V}}$CO_2_ occurred at exercise intensities that were similar to the intensity at which lactate threshold was observed and Mitchell and associates ([24]) reported that intravenous bicarbonate infusion prolonged exercise endurance and prevented changes in plasma [H^+^] or [HCO_3_^−^]. Moreover, it has been demonstrated that arterial pH during exercise above the RCP is primarily regulated by hyperventilation [11, 38]. The lack of “gold standard” measure of excess-$\dot{\mathrm{V}}$CO_2_ reduces the precision of the observed relationships and diminishes the power of the analyses, increasing the likelihood of type-2 error.

## Conclusions

This pilot study fulfilled its objective of assessing the feasibility of the current protocol in a future larger study investigating relationships between measures of $\dot{\mathrm{V}}$CO_2_ and performance fatigability. Based on its execution, the findings suggest that utilizing $\dot{\mathrm{V}}$CO_2_ measures may be a viable, and useful, addition or alternative to $\dot{\mathrm{V}}$O_2_ measures, warranting further study. While the current protocol was satisfactory, including in obtaining cardiopulmonary and performance fatigability measures as intended, modifications to the current protocol to consider in subsequent, larger studies may include use of an alternate mode or measure to enable control of work rate constancy during performance fatigability testing following initial CPET.

## Data Availability

The datasets used and/or analyzed during the current study are available from the corresponding author on reasonable request.

## Abbreviations

AT: Anaerobic threshold
AT-time: Time at the anaerobic threshold
AT-$\dot{\mathrm{V}}$O_2_: Oxygen consumption at the anaerobic threshold
BMI: Body mass index
CO_2_: Carbon dioxide
CPET: Cardiopulmonary exercise test
CWRT: Constant work rate test
EKG: Electrocardiogram
H^+^: Hydrogen ion
HR: Heart rate
Kt: Transition constant
excess-$\dot{\mathrm{V}}$CO_2_: Excess or non-metabolic carbon dioxide expiration
metabolic-$\dot{\mathrm{V}}$CO_2_: Metabolic carbon dioxide expiration
PFSS: Performance fatigability severity score
RER: Respiratory exchange ratio
RCP: Respiratory compensation point
t: Time
t-$\dot{\mathrm{V}}$CO_2_: Total $\dot{\mathrm{V}}$CO_2_
TD: Time delay
ORI: Oxidative response index
$\dot{\mathrm{V}}$CO_2_: Expired carbon dioxide
$$ \dot{\mathrm{V}} $$O_2_: Oxygen consumption
$\dot{\mathrm{V}}$O_2_-peak: Peak oxygen consumption
τ: Tau
Δ$\dot{\mathrm{V}}$O_2_: Amplitude change in oxygen consumption
10-MWT: 10-min walk test
